# Experienced career perspectives of nursing students and their supervisors in learning departments: a qualitative study

**DOI:** 10.1186/s12912-023-01479-3

**Published:** 2023-09-27

**Authors:** A. C. P. Boskma, F. W. Wolthuis, P. D. D. M. Roelofs, A. T. van Wijlen, J. E. van Schie, J. M. de Man- van Ginkel, E. J. Finnema

**Affiliations:** 1 Department of Health Science, University Medical Center Groningen, Groningen, Netherlands; 2 Nursing Science, Department of Gerontology and Geriatrics, Leiden University Medical Center, Leiden, Netherlands

**Keywords:** Learning department, Career perspective, Nurses, Nursing students

## Abstract

**Background:**

Due to the nursing shortage, positive work environments are needed to retain (student) nurses. More and attractive internships for students need to be ensured. In order to provide more internship places learning departments were developed, which are characterized by a buddy system and supervisors who coaches at least two students during a shift. Gaining knowledge about career perspectives and job satisfaction is essential within the context of learning departments, as both will contribute to quality and safety of care and will support lifelong learning. The current study aimed to investigate how nurses and nursing students working and learning in learning departments experience preconditions for career opportunities.

**Methods:**

Using a generic qualitative approach, semi-structured interviews were conducted through videocalls between March and April 2021 in the Netherlands. Inductive qualitative analysis based on ‘The Data Analysis Spiral’ was used.

**Results:**

Career perspective is explored among six students and seven nurses. Five main themes were generated for both nurses and students; (1) personal goals; (2) skills and self-efficacy; (3) mentoring; (4) job satisfaction; and (5) career perspectives. Within the five main themes, subcategories were developed from 198 codes related to career opportunities. Results show career perspective is experienced differently. For students, the requirements to experience career perspective seem largely existing, as learning departments fits with personal goals, increases self-efficacy and provide coaching mentoring. Students felt learning departments contributed positively to becoming more skilled in working independently and collaborating with fellow students. This resulted in students feeling well prepared for the future. Nurses’ career perspectives varied from wanting more personal development to experiencing opportunities due to having great colleagues, a challenging patient category, satisfaction from sharing knowledge and a decreasing physical workload. Nurses who had affinity with coaching students experience more career perspective on learning departments.

**Conclusion:**

Interviews provided in-depth insights. Interviews gave in-depth insight into the elements of learning departments that contribute to career perspectives of (student)nurses. The results can be used by nursing supervisors, teachers and policymakers to optimize nurses’ work environment, to eliminate leave intentions and improve quality of patientcare. The results should be taken into consideration when coaching students, developing manuals and implementing or optimizing learning departments.

Future research is recommended to investigate which tools/interventions are effective for nurses and other healthcare professionals to support career guidance.

## Introduction

A worldwide shortage of qualified nurses [1, 2] endangers healthcare [3]. Prolonged nurse shortages and high turnover increase nurse workload and place undue pressure on existing staff [4]. In 2025 125.000 extra nurses will be needed within Dutch healthcare [5]. Causes of nurses leaving are increasing complexity of healthcare due to the ageing population, insufficient numbers of nursing students, poor work environments and the physical and mental stress of the job [1]. In a situation where more nurses leave, rather than enter the profession, a nursing shortage arises [6].

Intention to leave is significantly associated with job satisfaction, work environment, quality, safety, staffing and tasks [1]. Another influencing aspect is the opportunity for career advancement (professional development and learning potential) for nurses [1], since experiencing career opportunities was shown to be a significant predictor of job satisfaction in various studies [7]. First, a cross-sectional study among 1538 registered nurses (RN’s) in New York showed that promotional opportunities and career orientation proved significant determining factors for job satisfaction [8]. Likewise, a cross-sectional study among 87 nurses from Washington showed the intent to leave and lack of job satisfaction appeared to be driven mainly by lack of opportunities for career growth and development, lack of extra remuneration for nurses who acquire additional degrees and certification, and lack of extra help during periods of high acuity [3].

Above, the career opportunities are described as an element focusing and influencing nurses’ retentions. Within the context of this study, the Netherlands, the Dutch government developed an action plan focusing on nursing student too [9, 10]. Sufficient internships and inspiring education should increase the number of students to address the nursing shortage in the end [9, 10]. Learning departments were developed based on factors for successful internship and best practice examples [11]. Calibris stated that learning departments are successful [10]. Pupils, supervisors and patients were enthusiastic and optimistic about the quality of these internships [10]. An University Medical Center (UMC) in the Netherlands designed their ‘best practice example’ learning department [11]. This learning department is mainly characterized by their manner of guidance, as one nursing supervisor who coaches at least two students during a shift [11].

Learning departments have a variety of nurses (e.g. senior nurses, in Dutch regieverpleegkundige), nursing students (e.g. with various education levels and years of experience) [11] and practical trainers. Practical trainers have a nursing background and are a contact point for students, they organize, coordinate and watch over the learning path of students.

Currently, a research project investigating work environment and job satisfaction is ongoing. One topic in this project is the development and evaluation of a format description for this specific set-up to anticipate on future-proof nursing care in a healthy learning and working environment with the specific aim to provide transferability of a best practice example [12].

Overall, improving career perspective is essential because high job satisfaction is associated with high-quality patientcare [1]. It potentially improves patients' perceptions of care quality and ensures an adequate nursing workforce [7]. Moreover, lifelong learning is increasingly seen as a precondition for sustainable employability [13, 14]. Focusing on students is just as important because 40% of recent graduated nurses leave the profession within two years and 27,5% leaves within one year [15]. Therefore it is essential to ensure sufficient attractive internships and focus on retention of recent graduates.

The current study explores the experienced career perspectives as part of a positive work environment within learning departments. In particular, we focus on career opportunities, because this is associated with quality and safety of patient care and intention to leave [1, 7, 16]. The relationships and predictors of career opportunities contribute to a more comprehensive understanding, which in turn may support the development of effective strategies to address the nursing shortage and increase patient care quality [17].

## Aim

The current study aimed to investigate how RN’s and nursing students working in learning departments experience the requirements for career opportunities.

## Methods

### Design

The study was generic qualitative in approach and descriptive in design to understand experiences from RN’s and student nurses’ point of view. Due to the explorative nature of the study aim, the choice for qualitative was appropriate [18]. A generic qualitative design is well-suited to explore participants’ experiences and offers the opportunity for participants to describe career perspectives and express their views in their own words [18]. The consolidated criteria for reporting qualitative studies (COREQ) is used to facilitate reporting of the results [19].

### Population

The study population consisted of nursing students and RN’s in three learning departments of one UMC in the Netherlands. The specialisms of the wards were cardiothoracic surgery, lung disease and rehabilitation. Participants were eligible when they were: (a)a RN working as supervisor in a learning department; (b)a student with experience in a learning department; (c)spoke Dutch. To encounter rich information the sampling strategy was purposive [20]. Participants were selected with a maximum variation on (a)years of work experience; (b)age [21–25]; (c)gender [21, 23] (d)hours work per week [22, 23]; (e)wards with different healthcare specialism [21]; (f)school institutes [23]; and (g)educational levels [21–25] because these factors are associated with career opportunities [21–25]. Maximum variation was applied to get access to perspectives from different nursing students and RN’s [20], and enhance the credibility of the data [26].

Current study focused on valuable and high-quality data to achieve data saturation. The aim was to recruit a minimum of twelve participants, since reaching saturation with this sample size seemed feasible [27]. Students had to have completed their internship in a learning department in order to provide rich data. Additionally, students who interned more than two years ago and RN’s who worked in learning departments more than two years ago were not approached to avoid recall bias. Ten intermediate vocational education students, eighteen bachelor students and three nursing teams (approximately 120 nurses) were approached. Reasons for declining participation are unknown.

### Procedures

Students’ were contacted via the internship office. Information, informed consent and an invitation to participate were sent by email. For ethical considerations see section ‘Ethics approval and consent to participate’. Two reminders were sent.

RN’s were recruited via the project leader of the learning departments and practical trainers. Additionally, the researcher visited two wards to introduce herself, provide study information and distribute posters. Due to a COVID-19 breakout on one ward introductory appointments were cancelled.

Recruitment took place from December 2020 till March 2021. Interviews were scheduled at the time most convenient for participants. The researcher was accessible by email for questions and comments. A pilot interview was conducted to practice and test the interview guide, hereafter the sequence of questions was adjusted. This interview was included to the analysis, as no substantive adaptions were made. Piloting the interview guide resulted in getting more used to the data and become more confident [20].

### Data collection

Semi-structured interviews were conducted between March and April 2021 by the first and second author. Due to COVID-19, interviews took place through videocalls (Microsoft Teams^©^), which are considered to be a proper alternative since differences in quality are sufficiently modest compared to face-to face interviews [27–31]. Of the videocalls, only audio tapes were used for analysis. Participants called in from home or work. An interview guide was used to ensure similar types of data from all informants was collected [20]. Themes covered the following areas: self-efficacy [32–35], skills and competences [21, 36], match personal goals [35, 37], job satisfaction [1, 35], work environment [1, 38], intention to leave the hospital and the profession [35], mentoring [34, 36, 38, 39], and funding and release time [33].

### Data analysis

Inductive qualitative analysis based on ‘The Data Analysis Spiral’ [18, 40] was used. Using Atlas.ti 8.4.25.0 (Scientific Software Development GmbH, Germany) transcripts were separated in meaningful segments related to the eight themes and labeled with codes. No transcript standards were used. Transcripts were coded independently by the first author(AB) and an external researcher, thereafter labels were discussed to reach consensus. Provisional outcomes were discussed within the research group, assumptions were formulated and the interview guide was amended. After open coding, fragments and codes were merged into sub categories and five main categories.

A back-and-forth movement between interviewing and analyzing was conducted to compare new insights and test insights in new rounds of data collection [20, 41]. Expectations and interim hypothesis were checked during interviews and the attainment of saturation could be made unadulterated [20, 41]. Additionally, theoretical sensitivity was reached by having knowledge about the subject and being aware of important concepts or issues that arose from the data [20]. An audit trial and memos were used to write down and link thoughts and methodological choices [18]. After analyzes member checking was performed. Results were sent to students and RN’s by e-mail. Written feedback was asked from participants to confirm assumptions, two participants provided feedback.

Additionally, the first author is a nurse with intrinsic motivations in the topic of the current study due to her own experiences and career choices. Being recognizable to participants can offers rapport and familiarity, but can also lead to colored perspectives. The first author was aware of this and she deliberately aimed to interview with an open, curious view. Furthermore, during interviews the second author was present. She was a good sparring partner to discuss interpretations since she specializes in educational science.

## Results

### Participants

**Fig. 1:**
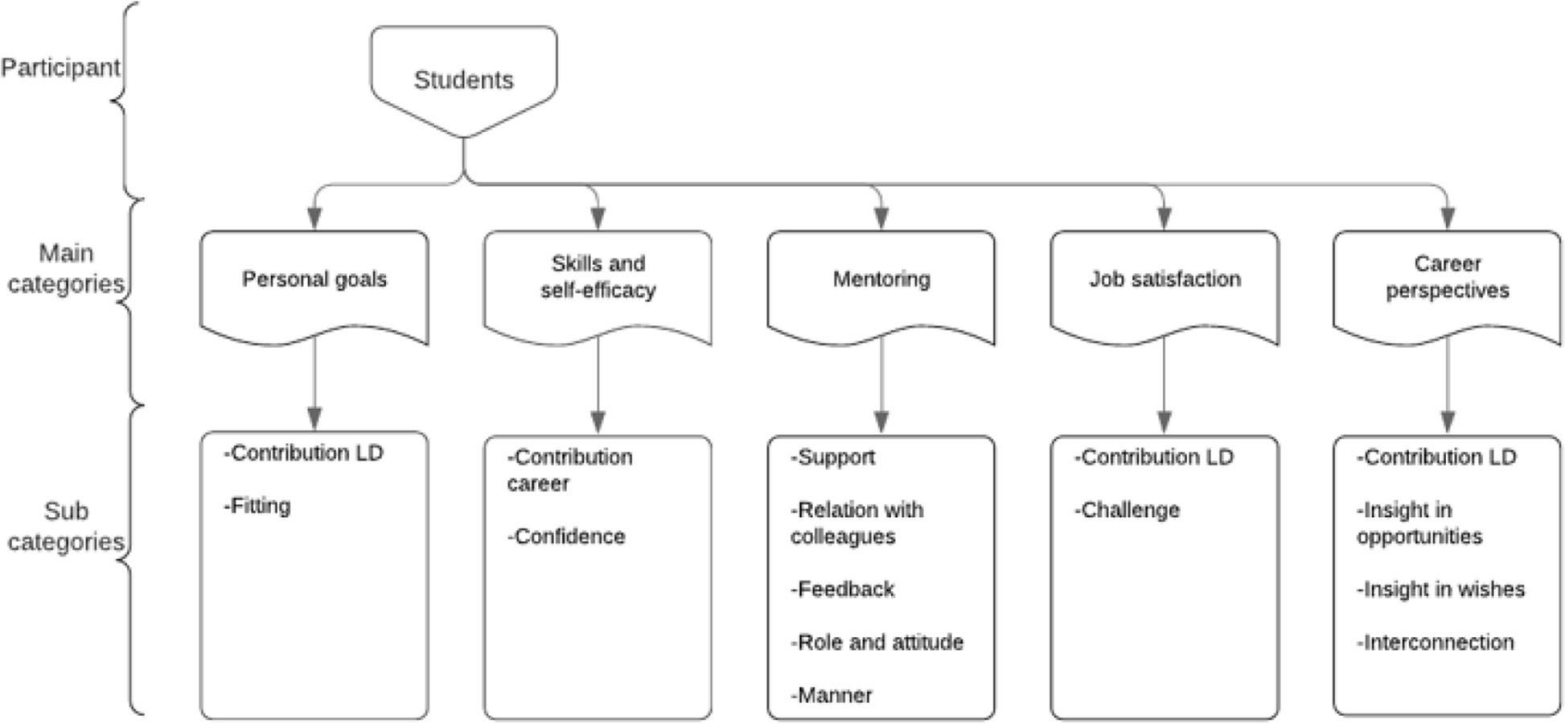
Coding scheme students. Note: LD: learning department

**Fig. 2:**
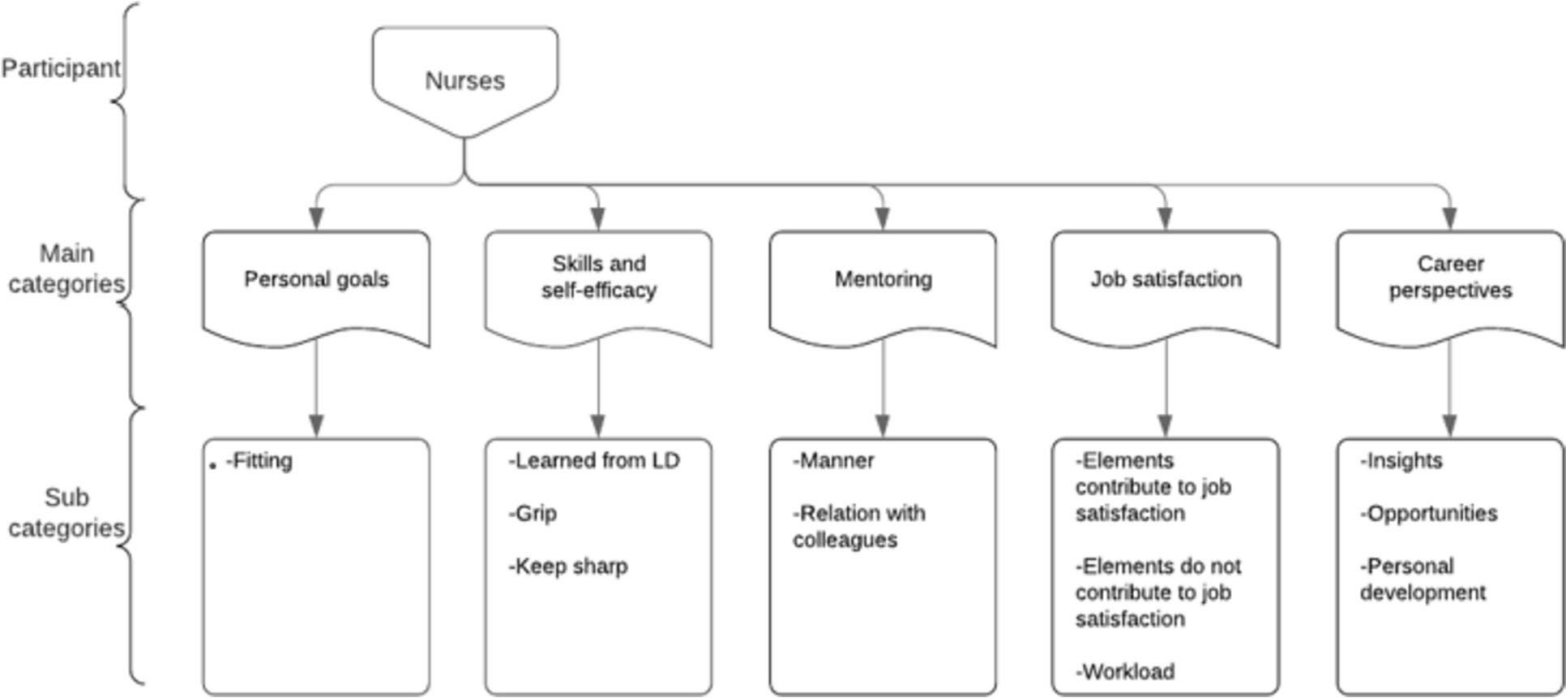
Coding scheme nurses. Note: LD: learning department

Participants are shown in Table 1. Six nursing students and seven RN’s were interviewed. For unknown reasons, one student recruited dropped out before the interview and seven students did not respond when approached with the request for an interview. Students’ mean age was 22,2 years (20–24), and RN’s mean age was 39,7 years (23–62). All the participants are female, which reflects the wider nursing population in the Netherlands which is primarily female. Within the sample, variation was achieved on education levels, ward specialism and years of work experiences. Interviews lasted between 47 and 61 min (mean 54 min) and were recorded and transcribed verbatim. Five main themes were generated for both nurses and students; (1) personal goals; (2) skills and self-efficacy; (3) mentoring; (4) job satisfaction; and (5) career perspectives. Within the five main themes, subcategories were developed from 198 codes related to career opportunities (Figs. 1 & 2). Saturation was achieved after 13 interviews, since new codes were no longer needed and formulated assumptions were confirmed. Although, this study was not aiming investigating differences between students and nurses, they emerged from analysis. Therefore, main themes will be further explored first for students and secondly for nurses in the following sections.

**Table 1:** Baseline characteristics

Baseline characteristics	Number of participants (%)
Total number of participants	13 (100)
Students	6 (46)
Registered nurses	7 (54)
Gender students	
Male	0 (0)
Female	6 (100)
Gender nurses	
Male	1 (14)
Female	6 (86)
Age in years students	
< 40	6 (100)
> 40	0 (0)
Mean	22,2
Age in years nurses	
< 40	(4) (57)
> 40	(3) (43)
Mean	39,7
Educational level students	
Vocational education	1 (17)
Bachelor	3 (50)
Vocational education + bachelor	2 (33)
Educational level nurses	
Inservice	3 (43)
Vocational education	2 (29)
Bachelor	1 (14)
Vocational education + bachelor	1 (14)
Ward specialism students	
Thorax	1 (17)
Lung	2 (33)
Revalidation	2 (33)
Thorax + revalidation	1 (17)
Ward specialism nurses	
Thorax	2 (29)
Lung	2 (29)
Revalidation	3 (43)
Years of work experiences nurses	
In total	
< 30	3 (43)
> 30	4 (57)
Mean	19,4
On the learning department	
< 15	4 (57)
≥ 15	3 (43)
Mean	11,6
Hours work per week nurses	
24	1 (14)
28	3 (43)
32	3 (43)

### Students

#### Personal goals

All students mentioned a learning goal-oriented environment when describing learning departments, since personal goals were considered and ample space was given. At the start of each shift, students make their learning goals for the shift known to their supervisors and fellow students. Reflection on learning goals generally takes places at the end of the shift. All students indicated this structure gave them insight into their own progress.

‘You’re very consciously working every day, you think about what will become my learning goal today, because you’ve to indicate what you want to learn that day. I think on a regular department you don’t do that so much, you think today I’ll just take care of patients and see what happens’ – Bachelor student.

#### Skills and self-efficacy

Many students indicated learning departments contributed to becoming more skilled in working independently and collaborating with fellow students. Being more competent in arguing care, assertiveness and leadership were also skills that students perceived as contributing to the career. All students felt learning departments contributed to gaining confidence in their competence.

‘I feel that I learned a lot, this gives you more self-confidence’ – Bachelor student

‘I found the buddy system, where you work with a felow student, a very positive part of the learning department. You learn how to work with your colleagues, similar to how you’ll do it later, discussing together and getting responsibility before going to your supervisor. You try to solve the problem instead of asking for help right away’ – Vocational education student.

#### Mentoring

Several students experienced support of fellow students, supervisors and practical trainers. Coaching was experienced as involved, safe, trusted, open and honest. Coaching is characterized by guiding and supporting students. The students are in de lead, which gives them more self-confidence and a realistic perspective on the authentic work environment. Some students described their mentoring as encouraging and stimulating which was stressful on the one hand, but provides a high learning return on the other hand. Mentoring was directed at becoming proficient in professional aspects, by observing, discussing the work, practicing, getting explanations and having actions checked/tested. A fixed training structure on the learning departments exists, which is similar for every student and was therefore perceived as not very personal but also as offering convenience and handles. According to students, career guidance was minimal. A few students indicated nurse supervisors were a role model or source of information. However, students also reported barely any conversations about career opportunities occurred. For example, some students said they were asked about future steps by supervisors but this was not a major subject within their interactions. Various students spoke of learning from fellow students by sharing knowledge and experiences. Students talking about career opportunities occasionally happed. As such, mentoring was mainly targeted on becoming competent in professional aspects, rather than focused on career counseling. Most students experienced mentoring positively which could influence the willingness to stay in the profession and hospital.

‘I don’t feel we worked on my career options in the way of talking about you can do that or what do you want after this’– Bachelor student

#### Job satisfaction

Most of the students experienced a welcoming environment. Collaboration in the learning department meant students challenge each other with questions and kept each other sharp. A reported downside of the high number of students on learning departments was that when patients arrived with interesting or complex care needs students had to share or ‘fight for’ the learning opportunities. Central to much of the students’ interviews was job satisfaction, resulting in them wanting to do this work later. They were happy with the internship placement.

#### Career perspective

Students felt learning departments provided insight in future intentions. All students stated working on learning departments resulted in a realistic idea of working as a nurse. Additionally, seeing different wards and specialisms could be helpful. Career opportunities are broad, but not always clear for students. Taking new career steps is especially relevant for students as for many, applying for jobs would be a logical next step after their (upcoming) graduation.

### Nurses

#### Personal goals

Nurses experienced learning departments as fitting their personal goals for the moment. Some nurses reported to expect to reach the developmental ceiling on learning departments after some time and were thinking about future-goals on other wards. Overall, learning departments are mostly set-up for educating student nurses. As such, students’ developmental goals play a bigger role on learning departments.

#### Skills and self-efficacy

Nurses described learning to coach and learning to give and receive feedback as contributing elements to their further career. Various nurses reported mentoring students contributed to their competence in arguing care. A few nurses experienced having less grip on patientcare because students provided this care. However, a majority of the nurses indicates that student questions ensures critical attitudes, a sharp eye and awareness of their actions.

‘They really just ask you why are you doing it like that and then you think, well, why? Then, you start thinking because, if you’ve been working for a long time you sometimes just act automatically, but they prevent you from that, because they keep you sharp’ – Inservice educated nurse.

#### Mentoring

Nurses explained career counseling was mainly organized during annual appraisals with senior nurses and/or head nurses. Counseling was experienced differently per ward. Some nurses experienced commitment and initiative from both sides (nurses and senior nurses/head nurses), others experienced the initiative had to come from themselves. Likewise, trust, safety and involvement within the team was experienced differently. Several nurses reported being satisfied, others described missing appreciation and support. Future plans and purposes are a part of their evaluation. When more or other opportunities were desirable, there was room for nurses to indicate this themselves.

‘The head nurse plans the annual appraisal, so you don’t have to think about that yourself. But if you want to have another conversation about your own development, that you’ve certain ideas about what I’d like to do that or that training, you can always discuss that with her. She’s always open to that’ – Inservice educated nurse.

#### Job satisfaction

For many nurses the challenging nature of the work and their colleagues were reasons to stay. Patient category was central for experiencing challenge in work and career perspective on the ward. Sharing knowledge and experiences with students resulted in satisfaction. Likewise, for some nurses, affinity with mentoring students was influential on their job satisfaction and staying in learning departments. Most nurses said they liked students’ fresh perspective. The ability to alternate between coaching students and direct patientcare appears relevant. Otherwise, less pleasure in work was indicated due to less patient contact. For nurses with physical complaints career perspectives can increase. The further the students’ internships progressed, the lower the physical workload seemed for nurses. Some nurses do not experience a reduction in workload, partly due to psychological burden of coordinating care and assessing school assignments.

‘I’d like to keep alternating between shifts per week on the learning department and not, so that you also continue to develop yourself’ – Vocational educated nurse

#### Career perspective

Nurses’ insight in opportunities varied. Insight is obtained by seeing other wards and specialisms, due to own efforts, conversations with colleagues and the hospital website. Career opportunities are felt in courses and training, which the organization facilitated on the one hand, but were expressed as nurses own responsibility on the other hand. Specific career opportunities related to learning departments are supervisor and practical trainer courses/positions. Other career opportunities are mostly associated with patient care, e.g. transplant nurse courses or nurse practitioner masters.

‘There’re many opportunities, on the ward patient care is complex and dynamic, so you can grow a lot there. Every year in your annual appraisal you are asked if you want to do something else, or go to a different department, which is very positive. If you’re assertive and you indicate that you need more challenge or are looking for something else, then you can get quite far with that’ – Vocational educated nurse.

## Discussion

In our study investigating how RN’s and nursing students working in learning departments experience the requirements for career opportunities, the thirteen interviews provided in-depth insights within various themes: personal goals, skills and self-efficacy, mentoring, job satisfaction and career perspectives.

Within the theme ‘personal goals’ interviews showed students’ developmental goals play a bigger role on learning departments. Students and nurses both experienced learning departments as fitting their personal goals, but some nurses reported to expect to reach the developmental ceiling after some time. For ‘skills and self-efficacy’ applies that students felt becoming more skilled in working independently and collaborating with fellow students. Nurses described learning to coach and learning to give and receive feedback as contributing elements to their further career. With regard to ‘mentoring’, the nurses guide the students. The students are in the lead, which gives them more self-confidence and a realistic perspective on the authentic work environment. For nurses their counseling was mainly organized during annual appraisals with senior nurses and/or head nurse. Central to much of the students’ interviews was job satisfaction, resulting in them wanting to do this work later. For many nurses the challenging nature of the work and their colleagues were reasons to stay. Taking new career steps is especially relevant for students as for many, applying for jobs would be a logical next step after their (upcoming) graduation. For nurses specific career opportunities related to learning departments are supervisor and practical trainer courses/positions.

Comparing results with existing literature, many similarities are found. First, students mentioned feeling well prepared for the future. Feeling confident about the future was reported and explained by previous literture [42–47]. For example, a dissonance between expectations and reality was described by students as resulting in a desire to leave the profession [42, 44, 45]. Further studies showed preparedness as satisfying [43, 46]. A possible explanation of these comparable outcomes might be the supervision which is part of all the context in which these studies took place(collaborative learning) [43, 46]. Every nurse coaches two students, as the buddy-system within current study. Some studies described the preparedness phenomenon as positive and a reason to stay in the profession [43, 46], other studies described the lack of the preparedness as a reason to leave the profession [42, 44, 45]. As such, our findings align with the literature.

Secondly, a lack of clear career opportunities was another result. While students mentioned realistic future representations and broad career opportunities, it was not always clear for students what next steps needed to be taken. Missing clear information about career prospects were reported earlier [42], which reinforces the current results.

Moreover, for nurses in learning departments, experiencing career opportunities seems not directly dependent on working on learning departments. Namely, the present study suggest nice colleagues were reasons for nurses to not leave the ward. The importance of supportive and empathetic relationships with colleagues appeared in various studies [45, 47, 48], and therefore the need of relatedness aligns with current study.

Finally, findings of the current study also support the significant positive correlation between willingness to stay and clinical stress [49], because the challenging demanding nature of the work and patient category were experienced by nurses as leading for experiencing challenge in work and career perspective on the ward. Additionally, the need for dynamics of nursing was previously stated as a factor affecting the career development of nurses [50].

### Strengths and limitations

The included heterogeneous group provided rich data from different perspectives which had strengthen the study. The achieved maximum variation will contribute to transferability [51]. Because a heterogeneous group was included there was room for various perspectives, improving intersubjectivity [51]. Intersubjectivity agreement was also improved by the independently coded transcripts by the first author, the second author and an external author [51]. Consensus was reached after discussion within the research team. Lastly, constant comparison was used, assumptions were formulated and member checking was conducted to optimize confirmability [51].

Some limitations need to be considered. Career perspective and opportunities are broad notions which have multiple interpretations. This can mean different participants referred to different notions when answering the interview questions. However, care was paid to this potential limitation by asking participants about their definition of career perspective during interviews. To strengthen interpretation triangulating in research design can be helpful. For example, combining observation research or action research can be conducted to give the results more rigor and quality. Second, career opportunities for students seem more logical after graduating, moreover the worldwide shortage of nursing could influence the experiences of opportunities. This can lead to students experiencing more career opportunities and focusing more on their next career steps. However, during interviews the researcher focused on contributions of learning departments to find relevant data to answer the research question. Further, most of the students were part of special hospital learning pathways aiming at interconnection, whereby a job was virtually guaranteed. Finally, including more men could have strengthened the study, although our sample represents the daily practice as nursing is a female-dominated occupation. Bachelor students were overrepresented, so possible education variation is minimal. However it is known that bachelor students increasingly attending advanced education [24]. However, a number of the responded bachelor students completed vocational education too.

### Implications for clinical practice and future research

The results can be used by nursing supervisors, teachers and policy makers to optimize work/learning department environments, deploy strategies to eliminate leave intentions and improve quality of patient care. The results should be taken into consideration when coaching students, develop manuals and implement or optimize learning departments. For example, career perspectives should be discussed with students from the perspective of the school program and the internship location. Topis to discuss could be: what attracts students in work, what are the career options and what steps need to be taken to achieve these options. Moreover, it is advisable to make an inventory of the wishes and needs of nurses by creating learning departments. Personnel who are interested in the educational side of nursing can be deliberately deployed. Future research is recommended to investigate which tools/interventions are effective for nurses and other healthcare professionals to support career guidance.

## Conclusion

The current study showed that career perspective was experienced differently by student nurses and registered nurses. Students mostly focus on questions such as ‘do I want to work in healthcare and this setting?’ and ‘am I competent enough?’, while nurses focus on the aspects of job satisfaction (colleagues, challenge, workload). This suggest that requirements to experience career perspective seems partially existing for students and RN’s in learning departments. Interviews gave in-depth insight into the elements of learning departments that contribute to career perspectives of (student)nurses. These insights can be used by nursing supervisors, teachers and policymakers to optimize nurses’ work environment, to eliminate leave intentions and improve quality of patientcare. The results should be taken into consideration when coaching students, developing manuals and implementing or optimizing learning departments. Future research is recommended to investigate which tools/interventions are effective for nurses and other healthcare professionals to support career guidance.

## Data Availability

The datasets generated and/or analyzed during the current study are not publicly available due privacy reasons but are available from the corresponding author on reasonable request.
